# BNN27, a 17-Spiroepoxy Steroid Derivative, Interacts With and Activates p75 Neurotrophin Receptor, Rescuing Cerebellar Granule Neurons from Apoptosis

**DOI:** 10.3389/fphar.2016.00512

**Published:** 2016-12-26

**Authors:** Iosif Pediaditakis, Alexandra Kourgiantaki, Kyriakos C. Prousis, Constantinos Potamitis, Kleanthis P. Xanthopoulos, Maria Zervou, Theodora Calogeropoulou, Ioannis Charalampopoulos, Achille Gravanis

**Affiliations:** ^1^Department of Pharmacology, School of Medicine, University of CreteHeraklion, Greece; ^2^Institute of Molecular Biology and Biotechnology, Foundation of Research and Technology-HellasHeraklion, Greece; ^3^Institute of Biology, Medicinal Chemistry and Biotechnology, National Hellenic Research FoundationAthens, Greece

**Keywords:** p75 neurotrophin receptor, DHEA, nerve growth factor (NGF), neurotrophin receptors signaling, neuronal apoptosis, steroid, STD NMR, molecular dynamics

## Abstract

Neurotrophin receptors mediate a plethora of signals affecting neuronal survival. The p75 pan-neurotrophin receptor controls neuronal cell fate after its selective activation by immature and mature isoforms of all neurotrophins. It also exerts pleiotropic effects interacting with a variety of ligands in different neuronal or non-neuronal cells. In the present study, we explored the biophysical and functional interactions of a blood-brain-barrier (BBB) permeable, C17-spiroepoxy steroid derivative, BNN27, with p75^NTR^ receptor. BNN27 was recently shown to bind to NGF high-affinity receptor, TrkA. We now tested the p75^NTR^-mediated effects of BNN27 in mouse Cerebellar Granule Neurons (CGNs), expressing p75^NTR^, but not TrkA receptors. Our findings show that BNN27 physically interacts with p75^NTR^ receptors in specific amino-residues of its extracellular domain, inducing the recruitment of p75^NTR^ receptor to its effector protein RIP2 and the simultaneous release of RhoGDI in primary neuronal cells. Activation of the p75^NTR^ receptor by BNN27 reverses serum deprivation-induced apoptosis of CGNs resulting in the decrease of the phosphorylation of pro-apoptotic JNK kinase and of the cleavage of Caspase-3, effects completely abolished in CGNs, isolated from p75^NTR^ null mice. In conclusion, BNN27 represents a lead molecule for the development of novel p75^NTR^ ligands, controlling specific p75^NTR^-mediated signaling of neuronal cell fate, with potential applications in therapeutics of neurodegenerative diseases and brain trauma.

## Introduction

Neurotrophic factors (NGF, BDNF and NT3/4) are large polypeptidic, secreted molecules that exert potent neuroprotective and neurogenic effects (Chao, 2003; Lu et al., 2005). Mature neurotrophins preferentially and selectively bind and activate the Trk receptors (Teng and Hempstead, 2004) while all members of neurotrophin family can also -albeit with lower affinity- bind to pan-neurotrophin p75 receptor. It is of note that neurotrophin immature isoforms (pro-neurotrophins) show high selectivity for p75^NTR^ (Lee et al., 2001; Dechant and Barde, 2002; Gentry et al., 2004; Nykjaer et al., 2004). The p75^NTR^ receptor is a member of the Tumor Necrosis Family Receptors (TNFR) superfamily and is mainly expressed during nervous system development but also after injury or degeneration of neuronal and glial cells (for a review, see Ibáñez and Simi, 2012). Despite the pro-apoptotic role of p75^NTR^, there is growing evidence that this receptor can also promote survival in specific types of neurons and other cells (DeFreitas et al., 2001; Massa et al., 2006; Meeker and Williams, 2015). The pro-survival effects of p75^NTR^ are largely dependent upon the operative ligands and the co-expression of other receptors that interact with p75^NTR^, like Trks or Sortilin (DeFreitas et al., 2001; Chao, 2003; Nykjaer et al., 2004). More specifically, activation of the transcription factor NFkappaB upon the recruitment of RIP2 protein to p75^NTR^, leads to survival signals on specific neurons (Carter et al., 1996; Vicario et al., 2015), while it simultaneously induces the release of RhoGDI from the receptor and the subsequent RhoA inactivation (Charalampopoulos et al., 2012). These p75^NTR^-mediated survival-signaling cascades are largely dependent upon mature neurotrophin binding to the receptor. On the contrary, the p75^NTR^-dependent regulation of cJun/JNK phosphorylation effectively induces cell death, although not as effectively as Caspase-3 (Bhakar et al., 2003). Cell death signals that originate from p75^NTR^ have been recently associated to other co-receptors, like sortilin, SorCS2 and SorL1 (members of the Vps10 family) (Nykjaer et al., 2004; Nykjaer and Willnow, 2012), and they are selectively activated upon binding of pro-neurotrophins to p75^NTR^. Furthermore, a plethora of other ligands can directly interact with p75^NTR^ receptors, such as Aβ-amyloid, myelin associated glycoprotein (MAG) or Nogo, leading to differential signaling in a cell-context manner (Wang et al., 2002; Wong et al., 2002; Yaar et al., 2002; Knowles et al., 2009).

We have previously reported that neurosteroid DHEA, produced by neurons, glia and the adrenals, binds to all Trk and p75^NTR^ receptors with Ki at nanomolar concentrations (Lazaridis et al., 2011; Pediaditakis et al., 2015), inducing cell survival (Charalampopoulos et al., 2004, 2008). DHEA can also activate classical steroid receptors, such as Androgen Receptor (AR) or Estrogen Receptors (ERα and ERβ), as well as a number of neurotransmitter receptors (Maurice et al., 2000; Chen et al., 2005). We have recently developed a novel C17-spiroepoxy analog of DHEA, BNN27, which selectively binds to and activates the NGF receptor TrkA, but not TrkB or TrkC (Pediaditakis et al., 2016). Modifications on C17 of BNN27 abolish its susceptibility to metabolic enzymes that convert DHEA to androgens or estrogens. Additionally, BNN27 has been shown to penetrate blood-brain-barrier (Bennett et al., 2016) and to not interact with AR or ERs in *in vitro* studies (Calogeropoulou et al., 2009; Pediaditakis et al., 2016).

The aim of the present study was to explore the ability of BNN27 to interact and activate the pan-neurotrophin p75^NTR^ receptors. We tested its biological effects in Cerebellar Granule Neurons (CGNs) of wt and p75^NTR^ null mice, an established cell model for the study of p75^NTR^ signaling. Indeed, CGNs express p75^NTR^ receptors and not the high affinity receptor for the NGF, TrkA (Courtney et al., 1997). They also express TrkB and TrkC receptors, providing the optimal substrate for evaluating the effects of other neurotrophins (BDNF and NT-3) compared to NGF. Cultured in the presence of serum, CGNs survive and show mature neuronal phenotype (Gallo et al., 1987; Balázs et al., 1988) Upon neurotrophin stimulation in serum-deprivation conditions, CGNs respond to survival signals: BDNF and NT-3 seem to rescue the cells through their specific Trk receptors, while NGF is inducing anti-apoptotic signals through the activation of p75^NTR^ (Minichiello and Klein, 1996; Courtney et al., 1997). We also evaluated the ability of BNN27 to interact with and activate p75^NTR^ receptors, focusing on the exact post-receptor cellular mechanisms by which it affects NGF-sensitive cell signaling, related to neuronal cell fate. Several physicochemical methods (NMR and pull-down assays with recombinant p75^NTR^) were also used to clarify the molecular interactions of BNN27 with p75^NTR^ receptors. Based on our findings we propose BNN27 as an effective lead molecule for the development of novel p75^NTR^ ligands, controlling specific p75^NTR^-mediated signaling of neuronal cell fate, with potential applications in therapeutics of neurodegenerative diseases and brain trauma.

## Materials and Methods

### Plasmids, Antibodies and Proteins

Plasmids expressing p75^NTR^, TrkA and TrkB were previously described Lazaridis et al. (2011) and Pediaditakis et al. (2015). p75^ΔECD^ and p75^C257A^ constructs were previously described by Jung et al. (2003) and Vilar et al. (2009), respectively. Normal expression of all constructs was verified by immunoblotting. The origin of antibodies was as follows: RIP2 (Cat. No. ADI-AAP-460; Enzo Life Sciences Farmingdale), RhoGDI (Cat. No. R3025; Sigma), p75^NTR^ for blotting [IB] (Cat. No. G3231; Promega), MC192 anti-p75^NTR^ for immunoprecipitation [IP] (Cat. No. MAB365R; Millipore), TrkA (Cat. No. 06-574; Millipore), TrkB (Cat. No. 4606; Cell Signaling), phospho-JNK (Cat. No. 4668; Cell Signaling), JNK (Cat. No. 9252; Cell Signaling), cleaved Caspase-3 (Cat. No. 9661; Cell Signaling); actin (Cat. No. A4700; Sigma). Secondary antibodies: horseradish peroxidase-conjugated anti-rabbit IgG (Cat. No. 65-6120; Invitrogen) and horseradish peroxidase-conjugated anti-mouse IgG (Cat. No. AP-124P; Millipore). Anti-rabbit-*R*-phycoerythrin conjugated (Cat. No. P9537; Sigma), anti-mouse-fluorescein conjugated (Cat. No. AP124F; Millipore), anti-rabbit Alexa Fluor 488 (Cat. No. A21206; Invitrogen), anti-rabbit Alexa Fluor 546 (Cat No. A10040; Invitrogen). NGF (Cat. No. 01-125) was purchased from Millipore. BDNF (Cat. No. P3595) and NT3 (Cat. No. P4433) were purchased from Novus.

### Animals

p75^NTR^ knockout mouse strain was obtained from Jacksons Laboratory (Lee et al., 1992) and maintained on a 12 h light/dark cycle with *ad libitum* access to food and water. Adult *het* mice were crossed and postnatal day 6 WT and KO pups were used for the isolation of cerebella after euthanasia in a CO_2_ chamber. Animal experimentation received the approval of Veterinary Directorate of Prefecture of Heraklion, Crete and was carried out in compliance with Greek Government guidelines and the guidelines of our ethics committee.

### Cell Culture

HEK293 cells were obtained from LGC Promochem and cultured under specific conditions for each cell line. MEFs were kindly provided from Dr C.F. Ibáñez (Karolinska Institutet) and cultured under standard conditions. HEK293 and MEF cells were transfected with the appropriate p75^NTR^ plasmids (wt, ΔECD, C257) by using TurboFect (Cat. No. R0531; Thermo Scientific, Rockford, IL, USA) according to manufacturer’s instructions. Transfected cells were typically used on the second day after transfection.

### Primary Cerebellar Granule Neurons Culture

Cerebellar granule neuron cultures were prepared as described previously, with some modifications (Selvakumar and Kilpatrick, 2013). Briefly, the cerebella of postnatal day 6 (P6) wt and p75^NTR^-knockout mice were isolated and digested with 0.25% trypsin (Sigma) for 15 min at 37°C. The enzyme was deactivated by adding DMEM/F12 (Sigma) medium containing 10% FBS (Gibco). The excess medium was removed and the remaining tissue was dissociated with 1 ml of culturing medium using the 1 ml pipette until cloudy. The cell suspension was filtered through a nylon mesh (pore size 70 μm) to remove any remaining big chunk of tissue. To obtain a culture enriched in CGNs the cell suspension was loaded onto a step gradient of 35 and 60% Percoll (GE Healthcare) and centrifuged at 1800 *g* for 20 min at room temperature. The upper interface was removed (containing astroglia, Purkinje cells and interneurons) and the interface, which contains the CGNs, was transferred to a 15 ml falcon tube. For extra enrichment the collected cell suspension was plated on poly-D-lysine coated petri dish for 20 min in a 5% CO_2_, 37°C incubator. Remaining astroglia adhere to the plate whereas granule neurons float. The supernatant was collected from the plate, centrifuged and re-suspended in the desire volume of the culturing DMEM/F12 medium supplemented with 2% B27 Supplement (Gibco), 0.6% D-glucose (Sigma), 1% GlutaMax (Gibco), 1% Penicillin/ Streptomycin (Gibco) and 250 μM KCl. Either half or 1/4 cerebellum was plated per well of a 12-well plate and 24-well plate, respectively.

### Immunoprecipitation and Western Blot

MEF cells were incubated with BNN27 (100 nM), DHEA (100 nM), NGF (100 ng/ml), E2 (100 nm) in the presence of serum free, washed twice with ice-cold PBS, and suspended in 400 μL cold lysis buffer containing 1% Nonidet P-40, 20 mM Tris (pH 7.4), and 137 mM NaCl, supplemented with protease inhibitors. Cleared lysates were pre-adsorbed with protein A-Sepharose beads (Amersham, Piscataway, NJ, USA) for 1 h at 4°C and IP with anti-p75^NTR^ overnight at 4°C. Protein A-Sepharose beads were incubated with the lysates for 2–4 h at 4°C with gentle shaking. For immunoblot (IB) analysis, the beads were suspended in sodium dodecyl sulfate-loading buffer and separated by SDS-PAGE. Proteins were transferred to nitrocellulose membranes and blotted with the corresponding antibodies. Then, the membranes were imaged with the ChemiDoc MP imager (Bio-Rad). ImageLab software version 4.1 (Bio-Rad) was used for image acquisition and densitometric analysis of the blots.

### Binding Assay

HEK293 cells were transfected with the cDNA expression plasmids coding for p75^NTR^ or p75^ΔECD^. Western blot inserts show the efficacy of transfection. Crude membrane fractions were isolated by differential centrifugation at 2,500 *g* (10 min at 4°C to remove unbroken cells and nuclei) and 100,000 *g* (1 h, at 4°C). A constant concentration of [^3^H]-DHEA (5 nM) was incubated with increasing concentrations of BNN27 (from 10^-12^ to 10^-6^M) in a final volume of 100 μl for 18 h at 4°C. Bound [^3^H]-DHEA was measured in scintillation fluid (SigmaFluor, Sigma) in a scintillation counter (Perkin Elmer, Foster City, CA, USA) with 60% efficiency for tritium.

### Pull Down and Saturation Transfer Difference (STD-NMR) Studies

#### Synthesis of BNN27 Conjugated to NovaPEG Amino Resin

BNN27 conjugated to NovaPEG amino resin was synthesized as previously described Pediaditakis et al. (2016).

### STD-NMR Experiments

The recombinant mouse NGFR/TNFRSF16-Fc chimera (also named p75 neurotrophin receptor) comprising the mouse NGFR ECD were purchased by R&D Systems, Inc. NGF (NGF 2.5S, mouse) was purchased by Millipore. D_2_O 99.9% from Euriso-Top was used for the PBS buffer.

NMR samples were prepared in Shigemi NMR tubes using D_2_O (99.9%) PBS buffer, pD = 7.4. The solutions of p75/BNN27 and NGF/BNN27 were prepared using a protein concentration of 0.6 μM and a ligand concentration of 60 μM, resulting in protein:ligand ratio of 1:100. Successive additions of NGF were applied to the solution of p75^NTR^/BNN27 at 1:1 up to 1:20 molar ratios of p75^NTR^:NGF. NMR spectra were acquired on a Agilent (Varian) 600 MHz NMR spectrometer at 25°C using a 1H{13C/15N} 5 mm PFG Automatable Triple Resonance probe. Experiments were run using the pulse sequences provided by the Varian BioPack library. The ^1^H STD NMR experiments were recorded with a spectral width of 12019 Hz, 8192 complex data points, and 4000 scans. Selective on-resonance irradiation frequency was set to -0.79 ppm for p75 and -0.94 ppm for NGF and the off-resonance irradiation frequency was applied at 30 ppm. The saturation scheme consisted of a train of 50 ms Gauss-shaped pulses separated by a 0.1 ms delay with total duration of 2.5 s. Subtraction of the on-resonance and off-resonance spectra was performed internally via phase cycling. Water suppression was achieved with excitation sculpting. Control spectra were recorded under identical conditions on samples containing only BNN27 to test for artifacts. The STD amplification factor was obtained by averaging the respective STD amplification factors for the C18 and C19 methyl group peaks of BNN27 in the studied solutions. The STD amplification factor is calculated according to the formula:

$$\text{STD amplification factor}=({\text{I}}_{0}-{\text{I}}_{\text{sat}})/{\text{I}}_{0}\times \text{ligand excess}$$

where (I_0_-I_sat_) corresponds to the STD intensity of one resonance signal and I_0_ is the corresponding intensity obtained from the off resonance spectrum or reference spectrum.

### *In silico* Modeling of BNN27 to p75^NTR^

The crystal complexes p75^NTR^/NGF 1:2 (pdb: 1SG1) and p75^NTR^/proNGF 2:2 (pdb: 3IJ2) were used for the molecular modeling studies. The crystal structure of p75^NTR^ dimeric complex with proNGF resolves the binding of the C-terminal mature region of mouse proNGF with p75^NTR^ ectodomain. Nine mutations were introduced at the mature proNGF monomers, namely, R9M, M37T, G40A, K50R, D60A, P61S, D65E, M92T, G94E, in order to model human-NGF in the p75^NTR^/proNGF 2:2 crystal structure (**Supplementary Figure 5**).

Binding site prediction was performed by applying SiteMap algorithm, Schrödinger Suite 2013, at the p75^NTR^ monomer (as obtained from pdb:1SG1 after removing NGF homodimer) and at the dimeric p75^NTR^ complexed with the modified proNGF, as described above.

The interactions of an ensemble of BNN27 molecules were investigated through Molecular Dynamics (MD) simulations in the cases of: p75^NTR^ alone (30 and 50 ns), p75^NTR^/NGF complex (200 ns) and the modified p75^NTR^/proNGF heterodimeric complex (150 ns).

Molecular Dynamics Simulations were performed with Schrodinger 3.8 using the OPLS3 force field. Each modeled system was solvated in an orthorhombic simulation buffer box of size 20 Å in each direction with TIP4P water molecules and the appropriate number of sodium counter-ions, randomly distributed in the aqueous phase. Before the production run, the model system was relaxed through a series of minimizations and short MD simulations were performed using Desmond default values. Production runs were performed in the NPT ensemble at 300 K and 1.01325 bar with periodic boundary conditions. The RESPA integrator was used with time step 2 fs for bonded and short-range interactions and 6 fs for long-range interactions. During equilibration the Berendsen thermostat and barostat were applied with coupling time constants of 100 ps and 1000 ps, respectively. The cutoff distance for the Coulombic interactions was set to 9 Å, while the long-range Coulombic interactions were calculated using the smooth Particle Mesh Ewald (PME) method with a tolerance of 1e-09.

### Cell Death Assay

BNN27 (100 nM), NGF (100 ng/ml), or BDNF (100 ng/ml) were added to cultures after 6 days CGNs *in vitro* and kept for an additional 16 h. During this period, CGNs were switched to KCl-free and serum-free medium. Finally, the culture was fixed with 4% Paraformaldehyde (PFA), permeabilised with 0.3% triton and blocked for non- specific epitopes with 5% Horse Serum (HS). Staining for β- tubulin+ was performed prior to TUNEL staining. The primary antibody (Cat. No. 801201, Biolegend) was incubated overnight at 4°C and the secondary anti-rabbit Alexa Fluor 488 (Cat. No. A21206; Invitrogen) for 1 h at RT. Subsequently, TUNEL was assessed using a kit from Roche following the manufacturer’s instructions and cultures were analyzed using a fluorescent Zeiss microscope. The number of TUNEL-positive cells co- localized with b-tubulin staining (yellowish nucleus) in each culture was counted using an objective (x40) from 10 visual fields for every condition. The percentage of TUNEL+ β- tubulin+/β- tubulin+ was calculated and the mean number was estimated for each condition from three independent experiments.

### RhoA Activity

RhoA activity was evaluated after 30 min neurotrophin stimulation using the RhoA G-Lisa kit from Cytoskeleton.

### Statistical Analysis

All results are reported as the mean ± SEM. Comparison of two groups was performed using an unpaired *t*-test. Statistical analyses were performed using GraphPrism, version 6 (GraphPad Software Inc.). A *P*-value of less than 0.05 was considered significant.

### Study Approval

All mouse experiments were approved by the Ethics Committee of the University Hospital of Heraklion, University of Crete, Faculty of Medicine and the Ethics Committee of the FORTH.

## Results

### BNN27 Directly Binds to p75^NTR^ Receptors

**FIGURE 1:**
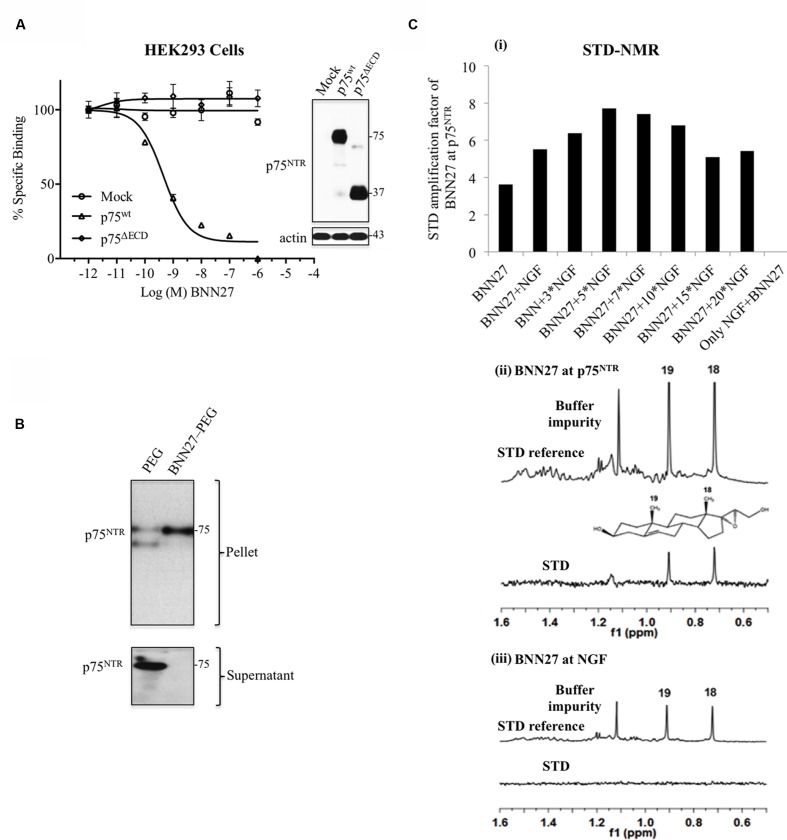
**BNN27 binds directly to p75^NTR^ receptor.** **(A)** Competition binding assays of [^3^H]-DHEA in the presence of increasing concentrations of BNN27, using membranes isolated from HEK293 cells transfected with the cDNAs of full-length p75^NTR^ or ECD-truncated p75^NTR^ΔECD receptors (Ki: mean ± SEM of five independent experiments). Right panel depicts efficacy of transfection, assessed by Western blots. **(B)** Immobilized BNN27 pulls down p75^NTR^ receptor. Covalently linked BNN27-7-*O*-(carboxymethyl) oxime (BNN27-7-CMO) to polyethylene glycol amino resin (NovaPEG amino resin) was incubated with recombinant p75^NTR^ proteins, then centrifuged. Precipitation experiments show Western blot analysis of pellets and supernatants with specific antibodies against p75^NTR^ proteins, as described in Section “Materials and Methods.” **(C, i)** STD-NMR revealed the interaction between BNN27 and p75^NTR^, further amplified upon NGF additions. No interaction of BNN27 with NGF alone was observed as suggested by the null STD amplification. **(ii,iii)** ^1^H STD-NMR and the corresponding STD-reference spectra. The binding of BNN27 at p75^NTR^ is evidenced by the presence of BNN27 methyls (18, 19) at the STD spectra; their absence in the case of NGF indicates the lack of BNN27-NGF interaction.

Firstly, we examined the ability of BNN27 to bind to p75^NTR^ receptor, performing heterologous competition binding assays, using tritiated DHEA. BNN27 effectively displaced the bound [^3^H]-DHEA on membranes isolated from HEK293^p75NTR^ transfectants (Ki: 3.9 ± 1.2 nM; **Figure 1A**). To evaluate the exact domains of p75^NTR^ required for BNN27 binding, we performed competition assays using membranes from HEK293 cells transfected with the p75^NTR^ mutant, lacking the entire ECD (with a smaller size as shown by immunoblot assays, **Figure 1A**, right panel), necessary for NGF binding (Jung et al., 2003). Our results showed that BNN27 was unable to bind to membranes isolated from HEK293p75^NTRΔECD^ mutants (**Figure 1A**), suggesting that the presence of the ECD is necessary for its binding to the receptor (**Figure 1A**).

We next assessed whether BNN27, covalently immobilized on NovaPEG resin, is able to bind directly to recombinant p75^NTR^, performing pull-down assays. Precipitation experiments and western blot analysis of precipitates with specific antibody against p75^NTR^ showed that immobilized BNN27 (BNN27-NovaPEG) effectively precipitated recombinant p75^NTR^ proteins, found in the pellet (**Figure 1B**). In contrast, there was no precipitation of recombinant p75^NTR^ in the absence of BNN27 (using only NovaPEG) (**Figure 1B**; the trace amount of p75^NTR^ seen in the pellet is due to residual supernatant). We also monitored the binding of BNN27 to p75^NTR^ by ^1^H STD-NMR spectroscopy. The presence of BNN27 resonance peaks (19Me and 18Me) in the STD spectrum (**Figure 1C, ii**) clearly reveals that BNN27 interacts with the recombinant p75^NTR^ receptor. On the other hand, the lack of the corresponding resonances in the NGF solution (**Figure 1C, iii**) indicates that BNN27 does not interact with the neurotrophin alone. The addition of NGF to the p75^NTR^ solutions induced an increase of the BNN27 STD amplification in a concentration dependent manner (**Figure 1C, i**). This behavior prompt**s** to the assumption of p75^NTR^/NGF gradual complexes formation in solution and evidences an enhancement of BNN27 binding at the heteromeric complex.

### *In silico* Studies of BNN27 Interactions With p75^NTR^ Receptors

It has been proposed that the functional profile of p75^NTR^ is related with the dimerized conformation of the receptor (Gong et al., 2008; Vilar et al., 2009). Our *in silico* strategy was driven by the absence of crystallographic data of the dimeric form of p75^NTR^ receptor in complex with the NGF homodimer. Thus, we have utilized the two most relevant crystal structures, the asymmetric complex with NGF (pdb:1SG1) and the symmetrical one with proNGF (pdb:3IJ2) modified accordingly to model NGF. Five potential binding sites were identified at the p75^NTR^ monomer as obtained after removing the NGF homodimer from the crystal complex 1SG1 (**Supplementary Figure 1**). Among them, only one (site **5**), was probed by MD simulations as the most potent to stabilize interactions with BNN27. Site **5** is located at the C-terminal loop of p75^NTR^ (ECD juxtamembrane CRD4 region) in the vicinity of the residues Cys152, Gln148, Thr140 and Val141, facing at the opposite side of the p75^NTR^/NGF interfacial region. The ligand uses only one of its polar edges either 3-OH or the substitution 21-OH functionalities to contact the protein surface (**Supplementary Figure 2**).

**FIGURE 2:**
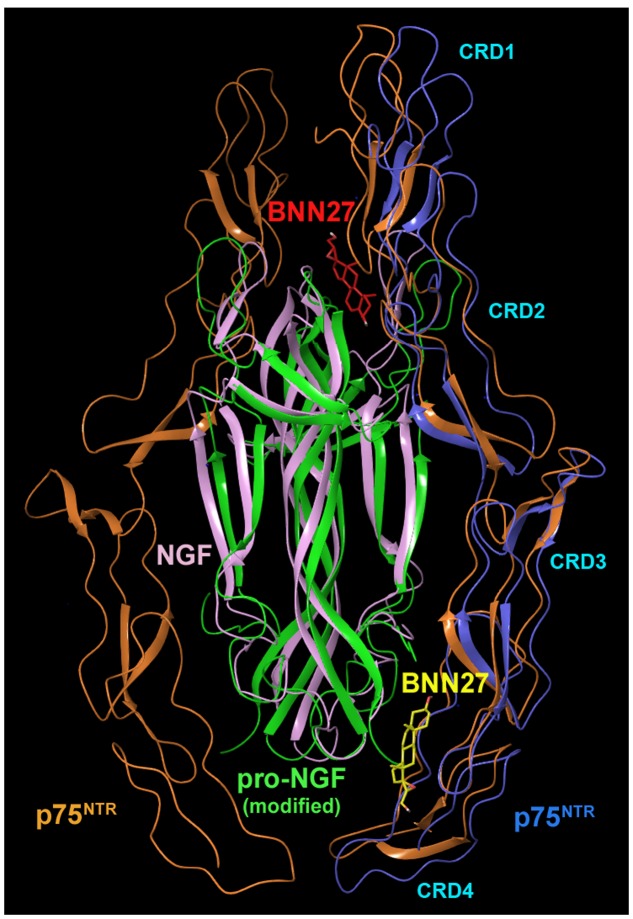
**Superimposition of representative MD simulations snapshots of BNN27 bound at p75^NTR^/NGF 2:1 and at p75^NTR^/mutated proNGF 2:2 complexes.** Superimposed frames of MD simulations performed at the p75^NTR^/NGF 1:2 and p75^NTR^/mutated proNGF 2:2 complexes with BNN27. The steroid analog (in yellow) is spontaneously inserted at the CRD4 interfacial region of p75^NTR^ (in blue)/NGF (in pink). At the modified p75^NTR^ (in brown)/mutated proNGF (in green), BNN27 (in red) spontaneously penetrates at the interfacial region located at p75^NTR^ CRD1-CRD2 junction.

Subsequently, we investigated the interactions of BNN27 with the complexed form of p75^NTR^ with NGF through a long exploratory MD run (200 ns). Interestingly, BNN27 initially located at site **5,** migrated toward the p75^NTR^/NGF interfacial region and stabilized interactions with crucial contact residues of the complex (NGF Arg^114^A and p75^NTR^ Cys^136^) (**Figure 2**; **Supplementary Figure 3**). The remaining BNN27 molecules, which were initially placed in the vicinity of the predicted binding sites of p75^NTR^, developed random contacts with the complex surface.

In a further step, we investigated the behavior of BNN27 with the heterodimeric p75^NTR^/proNGF 2:2 complex mutated as described above (see Materials and Methods) to model NGF homodimer. An ensemble of seven BNN27 molecules were placed in the neighboring of the predicted binding sites at the p75^NTR^/proNGF modified complex (data not shown) and MD simulations investigated the possibility of BNN27 to spontaneously penetrate the complex surface from the bulk water. Interestingly, simulations revealed the unprompted insertion of BNN27 already within the initial 40 ns, and its favorable harboring throughout the rest of the simulation time in an interfacial region located at the p75^NTR^ CRD1-CRD2 junction coinciding with the described NGF epitope as site I/patch 2 by (He and Garcia, 2004) (**Figure 2**). Specifically, BNN27 is “sandwiched” between the lipophilic residues NGF Phe^49^B and NGF Trp^99^B and contacts through H-bond with its 3-OH group p75^NTR^ Asp41 which additionally forms a salt bridge with NGF Lys^88^A, a conserved interaction also observed in both symmetric and asymmetric crystal structures of p75^NTR^ (**Supplementary Figure 4**).

In the symmetrical p75^NTR^/proNGF 2:2 complex the flexible hairpin loop L2 at the top of the mature region of proNGF dimer (residues 40–50) adopts an “open” state (Feng et al., 2010) which was not affected upon mutation to model human NGF. This conformation most probably favors the spontaneous insertion of the BNN27 ligand at the site I, patch 2 interfacial region located at the top of the complex. Moreover, (Pimenta et al., 2014) have proposed that the motion of the L2 loop influences the motion of the NGF L3 loop located at the lower part of the dimer (residues 60–70) and this may contribute to the unsuccessful approach of BNN27 to the hot spot identified from the MD run of the asymmetric complex of p75^NTR^ with NGF, in which the top NGF region adopts a more closed conformation.

### BNN27 Binding to p75^NTR^ Receptors Activates Downstream Signaling in MEF Cells

**FIGURE 3:**
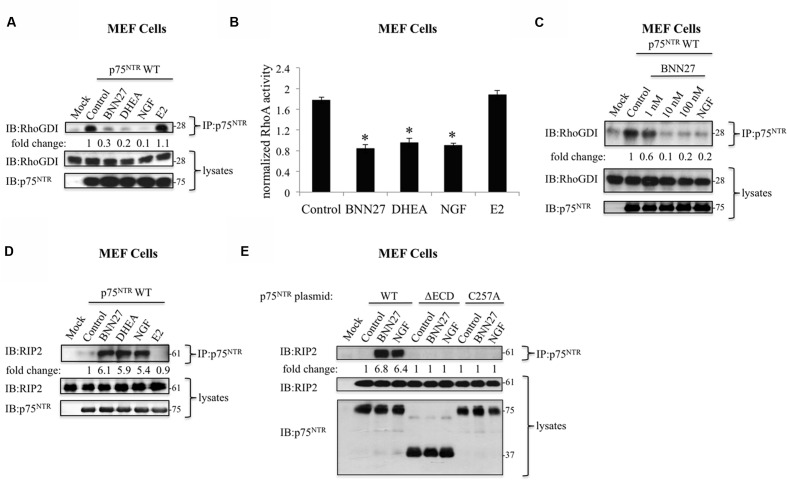
**BNN27 activates p75^NTR^ signaling.** **(A)** BNN27 induced the release of RhoGDI from p75^NTR^ in mouse embryonic fibroblasts (MEF cells). MEF cells were transfected with the plasmid cDNA of p75^NTR^ and control plasmid (Mock). Transfectants were exposed for 30 min with BNN27 (100 nM), DHEA (100 nM), NGF (100 ng/ml), or Estradiol (E2) (100 nM), and lysates were immunoprecipitated with p75^NTR^-specific antibodies, then immunoblotted with antibodies against RhoGDI. Total lysates were analyzed for p75^NTR^ expression by immunobloting. Fold change was calculated by densitometric scanning of RhoGDI (beads) signals normalized to RhoGDI (lysates) levels. Results are representative of three independent experiments. **(B)** Regulation of RhoA activity by BNN27 in MEF cells. Constitutively active RhoA protein (provided by the kit manufacturer) was used as positive control. Results are expressed as the mean of triplicate measurements ± SEM normalized to control. ^∗^*P* < 0.05 versus control. **(C)** BNN27 induced the release of RhoGDI from p75^NTR^ in a dose-dependent manner. MEF cells were transfected with the plasmid cDNA of p75^NTR^ and control plasmid (Mock). Transfectants were exposed for 30 min with various concentrations of BNN27 (1, 10, or 100 nM), or NGF (100 ng/ml), and lysates were immunoprecipitated with p75^NTR^-specific antibodies, then immunoblotted with antibodies against RhoGDI. Total lysates were analyzed for p75^NTR^ expression by immunobloting. Fold change was calculated by densitometric scanning of RhoGDI (beads) signals normalized to RhoGDI (lysates) levels. Results are representative of three independent experiments. **(D)** BNN27 induced the association of p75^NTR^ with its effectors RIP2 in MEF cells. MEF cells were transfected with the plasmid cDNA of p75^NTR^. Transfectants were exposed for 30 min to vector (Control), BNN27 (100 nM), DHEA (100 nM), NGF (100 ng/ml) or Estradiol (E2) (100 nM), and lysates were immunoprecipitated with p75^NTR^-specific antibodies, and then immunoblotted with antibodies against RIP2. Total lysates were analyzed for p75^NTR^ expression by immunobloting. Fold change was calculated by densitometric scanning of RIP2 (beads) signals normalized to RIP2 (lysates) levels. Similar results were obtained in three independent experiments. **(E)** Structure-function relationships in the interaction between BNN27 p75^NTR.^ MEF cells were transfected with the plasmid cDNAs of p75^NTR^ wt or p75^NTR^ mutants that lack the entire Extracellular Domain (ΔECD) or p75C257A and interactor RIP2. Transfectants were exposed for 30 min to vector (Control), BNN27 (100 nM), DHEA (100 nM), NGF (100 ng/ml) or E2 (100 nM), and lysates were immunoprecipitated with p75^NTR^-specific antibodies, and then immunoblotted with antibodies against RIP2. Total lysates were analyzed for p75^NTR^ expression by immunobloting. Fold change was calculated by densitometric scanning of RIP2 (beads) signals normalized to RIP2 (lysates) levels. Similar results were obtained in three independent experiments.

We tested the effectiveness of BNN27 to promote the interaction of p75^NTR^ with its effector proteins RhoGDI and RIP2, the two major signaling pathways activated by neurotrophin binding to p75^NTR^ (Khursigara et al., 2001; Yamashita and Tohyama, 2003). It is well documented that NGF induces the release of the intracellularly attached to the receptor, RhoGDI protein, which in its turn interacts with RhoA, blocking the activity of the latter (Yamashita and Tohyama, 2003). Moreover, the release of RhoGDI upon NGF-induced activation of p75^NTR^ was recently shown to be facilitated by the recruitment of another intracellular interactor, the RIP2 protein (Charalampopoulos et al., 2012). To investigate the effects of BNN27 on these specific signaling properties of p75^NTR^ receptor, we used primary MEFs, endogenously expressing RhoGDI and RIP2 but lacking p75^NTR^. Transiently transfected with p75^NTR^ plasmid MEF cell cultures were either untreated or incubated with 1-100 nM BNN27 for 30 min, then cell extracts were immunoprecipitated with an anti-p75^NTR^ antibody and the immunoprecipitates were analyzed by immunoblotting using an anti-RhoGDI antibody. As expected, endogenous RhoGDI was found to be associated with p75^NTR^ in transfected MEF cells and this interaction was decreased by NGF (**Figure 3A**). BNN27 similarly to DHEA and NGF induced the release of RhoGDI from p75^NTR^ (**Figure 3A**) and the subsequent significant decline of RhoA activity (**Figure 3B**). This effect of BNN27 on RhoGDI release was clearly observed at the concentrations of 10 and 100 nM, and less at 1 nM (**Figure 3C**). Interestingly, the steroid Estradiol (E2) was found to be ineffective (**Figures 3A,B**), indicating a steroid structure-specific effect.

Activation of p75^NTR^ signaling also involves the ligand dependent recruitment to the receptor cytoplasmic domain of specific cytoplasmic signaling molecules, such as RIP2. Thus, we examined whether p75^NTR^ in transfected MEF cells could interact with RIP2 in response to BNN27. Binding of the compound to p75^NTR^ elicited the recruitment of RIP2 to the receptor (**Figure 3D**), effect not shown with E_2_. NGF and DHEA treatment also induced the interaction between p75^NTR^ and RIP2 in transfected MEF cells (**Figure 3D**).

To further identify the domains of p75^NTR^ receptor interacting with BNN27, we examined the efficacy of BNN27 in recruiting the effector protein RIP2 on two p75^NTR^ mutants, p75^NTRΔECD^ lacking the entire ECD, and p75^NTRC257A^ lacking the ability to mediate RIP2 recruitment due to its low dimerization capacity (Vilar et al., 2009). Our findings show that the ECD of p75^NTR^ and its dimerized conformation are necessary for BNN27 to facilitate recruitment of RIP2 on p75^NTR^ receptors (**Figure 3E**), supporting our findings from the competition binding assays (**Figure 1A**).

### BNN27 Prevents Mouse Primary Cerebellar Granule Neurons from Apoptosis, in a p75^NTR^-Dependent Manner

The exact factors that enable p75^NTR^ to switch between its two opposite roles, mediator of pro-survival or apoptotic signaling, have not been identified with certainty. Indeed, p75^NTR^ receptor can trigger variant cellular effects due to its multiple signaling properties in a cell-context specific manner, tempospatial differences in ligand availability or co-receptors expression (Bronfman and Fainzilber, 2004; Vicario et al., 2015).

**FIGURE 4:**
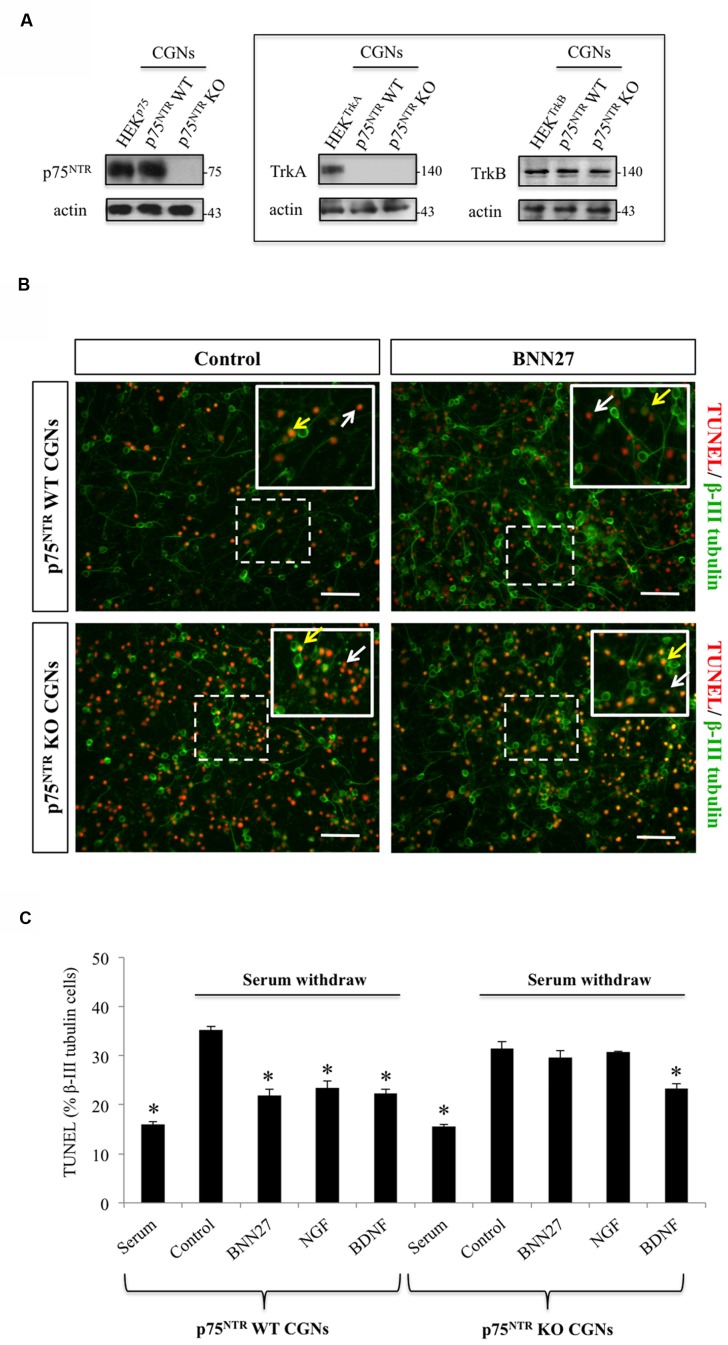
**BNN27 protects against apoptosis primary mouse cerebellar granule neurons.** **(A)** Neurotrophin receptors expression in cerebellar granule neuron cultures. Cerebellar granule neurons at 1 DIV express the pan-neurotropic receptor p75^NTR^ and TrkB, the selective receptor for BDNF, but they do not express TrkA. HEK293 cells transfected with the appropriate cDNA of neurotrophin receptors were used as positive controls. **(B)** Cell death in response to BNN27 treatment was assessed in p75^NTR^ wt (up-right) and p75^NTR^ knockout (KO) (down-right) cerebellar granule neurons (CGNs), identified by b-III tubulin immunostaining (green), using the TUNEL method (red). White arrows indicate TUNEL positive cells that do not express β-III tubulin (red nucleus) and thus represent a non- specific cell population whereas yellow arrows represent TUNEL and β-III tubulin double-positive cells (yellowish nucleus), which is the cell population that was count. Scale bar: 50 μm. **(C)** Quantification of TUNEL positive neurons CGN cultures (p75^NTR^ wt, left and p75^NTR^ KO, right) after 16 h serum withdrawal, incubation with BNN27, NGF or BDNF. Results are expressed as the mean ± SEM. (corrected to control) of three independent experiments, each performed in triplicate. ^∗^*P* < 0.05 versus control (Student’s *t*-test).

We tested the p75^NTR^-mediated effects of BNN27 in apoptotic signaling using the p75^NTR^-positive CGNs, lacking the expression of NGF high-affinity TrkA receptors. Consistent with previous reports (Minichiello and Klein, 1996; Courtney et al., 1997; Koshimizu et al., 2010), we confirmed that our CGNs cultures express p75^NTR^, TrkB, the selective receptor for BDNF, but not TrkA (**Figure 4A**). Furthermore, a weak but specific TrkC-positive immunoreactivity was also shown in CGNs (data not shown). BNN27 is activating both TrkA and p75^NTR^ but not TrkB or TrkC receptors (Pediaditakis et al., 2016). Thus, CGNs represent a suitable model of neuronal cells to investigate the actions of BNN27 specifically through p75^NTR^ receptors.

We examined the ability of BNN27 to control apoptotic cell death, of primary cultures of CGNs induced by serum deprivation, and assessed by TUNEL assay. Pilot time-course experiments revealed that the maximum apoptotic cell death upon serum deprivation was occurring at 24 h (data not shown). Thus, CGNs cultures isolated from wt and p75^NTR^-KO mice were exposed to 100 nM BNN27 for 24 h, fixed and stained for TUNEL assay (indicator of late-apoptotis) and the neuronal marker b-III tubulin (**Figure 4B**). BNN27 effectively protected wt-CGNs from apoptosis. Interestingly, the protective effects of both NGF and of BNN27 were completely abolished in p75^NTR^ –KO-CGNs (**Figure 4C**). On the contrary, the anti-apoptotic effect of BDNF was still present in p75^NTR^ –KO-CGNs, most probably mediated by the TrkB receptors. These findings suggest that the neuroprotective actions of BNN27 in CGNs are exclusively mediated by p75^NTR^, mimicking the well-described pro-survival effects of NGF in this neuronal type.

### BNN27 Propagates p75^NTR^-Depenedent Pro-survival Signaling in Primary Cerebellar Granule Neurons

The p75^NTR^ receptor has been shown to engage multiple signaling pathways, including the cell death-related JNK and Caspase-3 (Bhakar et al., 2003), as well as the pro-survival NFκ B, in association with its upstream TRAF6 and RIP2 effector proteins (Khursigara et al., 1999, 2001).

**FIGURE 5:**
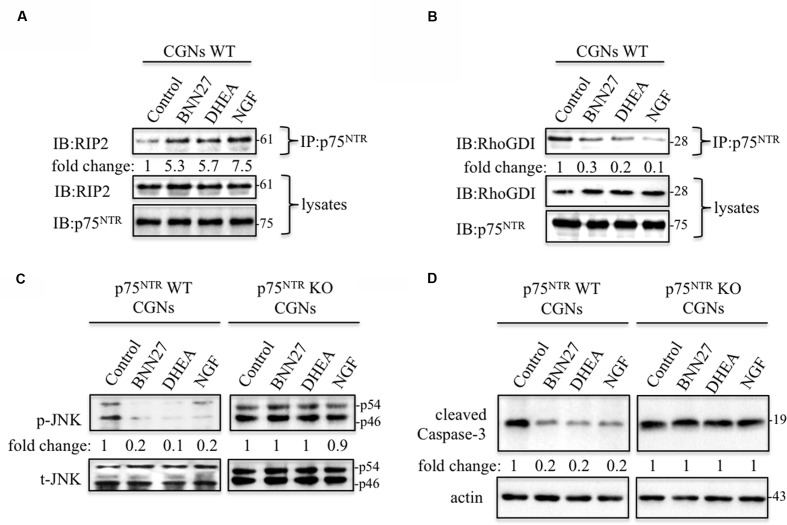
**BNN27 decreases cell death signaling in primary mouse cerebellar granule neurons.** **(A)** Co-immunoprecipitation of RIP2 with p75^NTR^ in CGNs isolated from p75^NTR^ wt mice. Incubation of CGNs for 20 min with BNN27, DHEA and NGF induce the recruitment of RIP2 from p75^NTR^. Fold change was calculated by densitometric scanning of RIP2 (beads) signals normalized to RIP2 (lysates) levels. Similar results were obtained in three independent experiments. **(B)** Co-immunoprecipitation of RhoGDI with p75^NTR^ in CGNs isolated from p75^NTR^ wt mice. Incubation of CGNs for 30 min with BNN27, DHEA and NGF induce the release of RhoGDI from p75^NTR^. Fold change was calculated by densitometric scanning of RhoGDI (beads) signals normalized to RhoGDI (lysates) levels. Similar results were obtained in three independent experiments. **(C)** Levels of phosphorylated c-jun kinase (p-JNK) in CGNs isolated from wt and p75^NTR^ KO mice. BNN27, DHEA or NGF Fold change was calculated by densitometric scanning of phospho-JNK signals normalized to total JNK levels. Results are representative of three experiments. **(D)** Activated Caspase-3 in CGNs, isolated from wild-type (WT) and p75^NTR^ knockout (KO) mice. After 16 h serum withdrawal treatment. Fold change was calculated by densitometric scanning of cleaved-Caspase-3 signals normalized to actin levels.

Thus, we performed co-immunoprecipitation studies to assess the effects of BNN27 in the above p75^NTR^ related signaling pathways in primary CGNs, starting with the recruitment of RIP2 to p75^NTR^, an important condition for the activation of pro-survival NFκB pathway. BNN27 rapidly and in an NGF/DHEA-similar manner, induced RIP2 recruitment to p75^NTR^ in CGNs (**Figure 5A**). In previous studies we have shown that ligand-dependent RIP2 recruitment is structurally linked to the release of RhoGDI protein, since they both compete for the same amino acid residues in the Death Domain (DD) of p75^NTR^ receptor (Charalampopoulos et al., 2012). Based on this observation, we tested the ability of BNN27 to mimic NGF and DHEA in releasing RhoGDI from p75^NTR^ receptor. As suggested by the RIP2 recruitment experiments, BNN27 was also effective to release RhoGDI from the DD of p75^NTR^ receptor (**Figure 5B**).

We finally investigated the ability of BNN27 to induce phosphorylation of pro-death c-jun kinase (JNK) in cultured CGNs, isolated from wt and p75^NTR^-KO mice. As expected, levels of active JNK were elevated in CGNs cultured under serum-deprived conditions. Incubation with 100 nM BNN27 for 30 min decreased the activity of JNK by 80% in the aforementioned conditions (**Figure 5C**). The response to BNN27 was p75^NTR^-dependent, since it was fully abolished in CGNs, isolated from p75^NTR^-KO mice (**Figure 5C**). Furthermore, BNN27 attenuated the activation/cleavage of pro-apoptotic Caspase-3 in CGNs isolated from wt mice, (in agreement with the TUNEL assay results, **Figures 4B,C**), an effect which was also absent in CGNs from p75^NTR^KO-mice (**Figure 5D**). In addition, DHEA, similarly to BNN27, is not affecting JNK or Capsase-3 activity in p75KO-derived CGNs, clearly indicating that it also acts exclusively via p75^NTR^. These results confirm our previous observations that BNN27 and DHEA selectively activate the p75^NTR^ receptor but not TrkB (Pediaditakis et al., 2015, 2016).

## Discussion

The low-affinity receptor of NGF, p75^NTR^, was firstly identified in Chao et al. (1986) and Johnson et al. (1986), as a member of the TNF receptors superfamily. It bears a DD, affording its function as a death receptor. Many neuronal cells during nervous system development or after injury are undergoing apoptosis through activation of p75^NTR^ in a cell-autonomous or non-autonomous manner with elevation of its expression (reviewed in Ibáñez and Simi, 2012; Meeker and Williams, 2015). Interestingly, p75 exon-3 knockout mice, showed reduced cell death in the retina (Lee et al., 1992). The pro-death functions of p75^NTR^ are mainly activated by immature forms of neurotrophins (pro-neurotrophins) and parallel cross-talk with another membrane co-receptor, sortilin (Nykjaer et al., 2004). Mature neurotrophins can also induce p75^NTR^-dependent cell death, but these actions oppose their effects through their high-affinity specific receptors, the pro-survival Trk receptors. It thus appears that neurotrophin signaling properties are rather complicated and each neurotrophin receptor presents a context-dependent activity profile. Indeed, even if the knowledge on the diverse functions of this receptor is still weak, p75^NTR^-mediated actions include cell survival or death, regulation of neuronal precursor proliferation and inhibition of neurite outgrowth, synaptic pruning and long-term depression, depending upon the co-expression of Trk receptors, co-receptors like sortilin or Nogo, various intracellular signaling adaptor proteins like RIP2, TRAF6 or RhoGDI, and finally a plethora of potential ligands, like immature and mature neurotrophins, Aβ peptide, or myelin derivatives (reviewed by Lu et al., 2005; Schor, 2005; Meeker and Williams, 2015). The tempo-spatial expression of p75^NTR^ receptors in many CNS structures as the basal forebrain, striatum and cerebellum in normal and pathological conditions, has highlighted the importance of this receptor in neurodegenerative conditions. During the last decade p75^NTR^ has emerged as a potential therapeutic target for many neurodegenerative disorders (reviewed in Shu et al., 2015). Various pharmacological strategies were specifically targeting p75^NTR^, aiming to constrain its multiple responses in a cell-context manner. Endogenous neurotrophins are lacking a favorable pharmacokinetic profile, because of their polypeptidic nature and their inability to penetrate the BBB. Therefore efforts for developing small molecules have been recently undertaken (reviewed in Longo and Massa, 2013). In the present study we propose a highly lipophilic, BBB-permeable synthetic steroid as a potential lead molecule to develop p75^NTR^ receptor ligands.

Our findings provide biochemical and biophysical evidence that the synthetic 17-spiroepoxy derivative of neurosteroid DHEA, BNN27, effectively interacts with p75^NTR^ receptors. BNN27 aims at surpassing the limited clinical value of DHEA and its profile as a non-specific multi-receptor activator and a precursor for the biosynthesis of androgens and estrogens (Calogeropoulou et al., 2009). Indeed, neurosteroids DHEA and DHEA-S, produced within the brain, effectively protect neurons against apoptosis (Charalampopoulos et al., 2004). Surprisingly, DHEA was shown to bind and activate all invertebrate and vertebrate Trk neurotrophin receptors as well as the p75^NTR^ pan-neurotrophin receptor in various neuronal cell types, representing most probably an ancestral in evolution neurotrophic factor (Lazaridis et al., 2011; Pediaditakis et al., 2015). However, the potential clinical use of DHEA, as a long-term neuroprotective therapeutic is compromised by its multiple secondary effects via its binding to various steroid (estrogen receptors alpha and beta, ARs) and neurotransmitter receptors (GABA_A_, NMDA, and sigma1 receptors) (Charalampopoulos et al., 2008). Last but not least, DHEA holds a central role in steroidogenesis, as a precursor steroid in the biosynthesis of androgens and estrogens (Charalampopoulos et al., 2008). It is of note that recent studies associate DHEA to an increased prevalence of hormone-dependent tumors, particularly to genetically predisposed patients (Fourkala et al., 2012). Thus, BNN27 represents a more specific, with potentially less secondary effects neuroprotective agent, showing specific binding to only TrkA and p75^NTR^ receptors, and being deprived of estrogenic and androgenic actions, unable to bind to and activate steroid receptors (Calogeropoulou et al., 2009; Pediaditakis et al., 2016).

We have recently shown that in neuronal populations expressing both TrkA and p75^NTR^, BNN27 is exhibiting TrkA-mediated, pro-survival effects, acting as a NGF mimetic, without affecting TrkB or TrkC receptors (Pediaditakis et al., 2016). However, the study of the effects of BNN27 through the p75^NTR^ receptor was challenging in these neuronal cell types, since p75^NTR^ may act synergistically, antagonistically or independently of TrkA receptors. In the present study, we sought to explore the decoupling of the effects of BNN27 through TrkA and p75^NTR^ receptors, using neuronal cells expressing only one of these receptors, p75^NTR^.

Competition binding assays in cells transfected with p75^NTR^ and lacking Trk receptors, pull-down experiments with recombinant p75^NTR^ and PEG-immobilized BNN27, as well as STD-NMR studies and *in silico* studies clearly show that BNN27 is capable to physically interact with p75^NTR^, and this interaction requires the ECD of the receptor. Furthermore, STD-NMR revealed that the presence of NGF at the p75^NTR^/BNN27 solution induces an enhancement of BNN27 interactions with the formed p75^NTR^/NGF complex. Molecular dynamic simulations confirmed the interaction of BNN27 with the p75^NTR^ receptor as a monomer probing the juxtamembrane CRD4 region as the most accessible for BNN27. Moreover, *in silico* studies revealed that BNN27 interacts fruitfully with the complexes p75^NTR^/NGF 1:2 and p75^NTR^/proNGF 2:2 mutated to model human-NGF homodimer. Specifically, BNN27 was spontaneously inserted at the interfacial region of p75^NTR^/NGF asymmetric complex close to the juxtamembrane region while in the case of the symmetric p75^NTR^/mutated proNGF complex the ligand was accommodated at a hydrophobic pocket (site I, patch 2 region) located at p75 CRD1-CRD2 junction. Thus, molecular dynamics simulations confirmed the interaction of BNN27 with p75^NTR^:NGF complex as was evidenced by STD-NMR data. The proposed hot spots are not conclusive given the limitations imposed by the flexibility of the NGF loops L2 and L3, however, in the absence of additional crystallographic data they could assist the efforts toward optimized ligands.

As a cellular system to assess the p75^NTR^-dependent effects of BNN27, we have chosen the primary neuronal cultures of mouse CGNs. These neurons express p75^NTR^, TrkB and TrkC, but not TrkA. It is well described by many studies (Minichiello and Klein, 1996; Courtney et al., 1997), that BDNF and NT-3, ligands for TrkB and TrkC respectively, protect CGNs against toxic stimuli. Additionally, proBDNF acts as a p75^NTR^- mediated apoptotic signal in this type of neurons (Koshimizu et al., 2010).

Surprisingly, in addition to promoting apoptosis, p75^NTR^ is known to also favor neuronal survival. Indeed, the ability of p75^NTR^ to enhance neuronal or glial survival is shown by multiple studies (DeFreitas et al., 2001; Hannila and Kawaja, 2005; Wehner et al., 2016), been associated to the activation of pro-survival transcription factor NFκB (Hamanoue et al., 1999). Interestingly, binding of RIP2 to p75^NTR^ leads to NFκB activation (Khursigara et al., 2001). Recent studies have also indicated that RIP2 recruitment facilitates the release of RhoGDI by p75^NTR^, competing for common residues at the DD of the receptor (Charalampopoulos et al., 2012). The release of RhoGDI blocks the down-stream RhoA protein. The Rho family of GTPases belongs to the Ras superfamily of low molecular weight (21 kDa) guanine nucleotide binding proteins, and is associated to pro-apoptotic processes (Dubreuil et al., 2003; Sanno et al., 2010).

Genetic and pharmacological studies have documented the different signaling properties of the p75^NTR^ receptor, depending upon differences in proteolytic cleavage of the receptor (Vicario et al., 2015), in intracellular machinery (Lu et al., 2005) and in co-expression of various co-receptors (Lee et al., 2001; Wang et al., 2002; Knowles et al., 2009). More specifically in the CGNs neuronal population, ligand-dependent activation of p75^NTR^ was unable to induce cell death, due to a strong activation of pro-survival NFκB that prevailed the apoptosis signal. On the contrary, hippocampal neurons exhibited increased apoptosis upon p75^NTR^ activation because of their inability to adequately activate NFκB (Vicario et al., 2015). In the present study, we describe the efficacy of BNN27 to prevent apoptosis of CGN neurons, in a p75^NTR^-mediated manner. These neuroprotective effects of BNN27 are afforded by the activation of RIP2 and the concomitant release from p75^NTR^ receptor of RhoGDI, the blockade of down-stream pro-apoptotic RhoA and the activation of pro-survival transcription factor NFκB.

**FIGURE 6:**
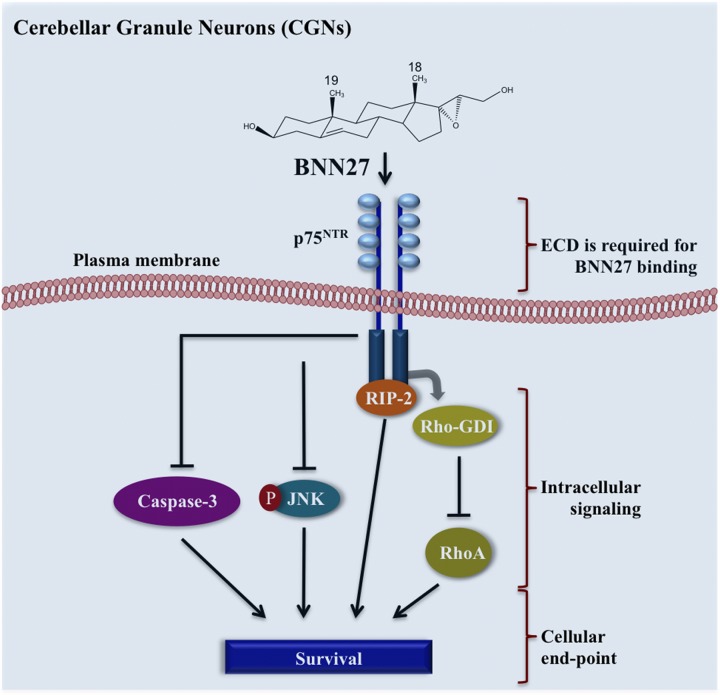
**Schematic illustration of the pro-survival effects of BNN27 in a p75^NTR^-mediated manner in primary mouse cerebellar granule neurons.** BNN27 activates p75^NTR^ receptors, leading to the control of pro-survival RhoGDI and RIP2 effectors while in parallel attenuating the activation of pro-apoptotic JNK and Caspase-3 factors.

In addition to the pro-survival signals that BNN27 induces through p75^NTR^ receptors, it inhibits in parallel specific pro-apoptotic signals. Previous studies have reported that overexpression of p75^NTR^ in primary cortical neurons, PC12 cells, or glioma cells leads to the activation of both JNK kinase and apoptotic Caspase-3 (Gu et al., 1999; Wang et al., 2001; Harrington et al., 2002; Bhakar et al., 2003). We now report that BNN27 is effective in attenuating both the phosphorylation/activation of the pro-apoptotic JNK and the cleavage of apoptotic executor Caspase-3, thus enhancing CGNs survival in parallel with its beneficial effects on pro-survival signaling. The inhibitory effect of BNN27 on pro-apoptotic JNK and Caspase-3 is mediated by p75^NTR^ receptors since it is totally abolished in CGNs, isolated from p75^NTR^-KO mice. These findings strongly suggest that specific neurons that express p75^NTR^ but not TrkA, respond to BNN27 by enhancing their pro-survival signaling upon growth factors derivation, and this effect is dependent on p75^NTR^ expression (**Figure 6**). On the other hand, BDNF induces survival in the same p75^NTR^-lacking neurons, supporting our interpretation that BDNF, presumably acts via the TrkB receptor.

The p75^NTR^ receptor emerged recently as a new therapeutic target for various conditions, including neurodegenerative diseases, brain trauma and neuronflammation. Indeed, identification through *in silico* screening of the first non-peptide small-molecule ligands modulating p75^NTR^ signaling, generated several alternative strategies, mainly elucidated from other receptors studies (reviewed in Longo and Massa, 2013). These new synthetic ligands target the induction or inhibition of dimerization/activation of p75^NTR^, affecting its membrane conformation. It is worth noticing that p75^NTR^ is a multidomain, multifunctional receptor, interacting with a plethora of ligands, most probably through different domains, some of which may principally mediate binding, whereas others recognize specific ligand domains, differentially affecting receptor conformation. The limited size of small molecules restrain their ability to fully emulate the multiple set of multi-domain receptor interactions of polypeptidic, native neurotrophins. Our synthetic compound, BNN27, represents a new class of potential ligands for p75^NTR^ receptor, with agonist actions, depending upon the cell context. Its lipophilic structure offers important pharmacological advantages, conferring BBB penetration (Bennett et al., 2016). Additionally, it easily integrates into the membrane lipophilic environment, reaching very important trans-membrane residues of the receptor, including the Cysteine-257, known as the residue with a pivotal role for the dimerization of the receptor (Vilar et al., 2009). The presence of cell-specific membrane co-receptors (TrkA and others) may locally modify the functions of BNN27 through p75^NTR^ and, consequently, the effects of p75^NTR^-mediated signaling, allowing a broad range of actions, potentially distinct from these of native neurotrophins. The knowledge emerging from studies on the interaction of BNN27 with p75^NTR^ may facilitate the design of molecules with tailored therapeutic properties, developing drugable disease-specific compounds.

## Author Contributions

AG and IC conceived and supervised the project. AG, IC, TC, and IP designed the experiments, interpreted data, drafted and revised the manuscript. IP and AK performed most of the experiments and analyzed the data. AK set up and used primary neuronal cultures. KP, synthesized BNN27 covalently immobilized on NovaPeg resin, CP, KX, MZ performed STD NMR experiments and molecular modeling studies. All authors approved the version that was submitted.

## Conflict of Interest Statement

AG is the co-founder of spin-off Bionature EA LTD, proprietary of compound BNN27 (patented with the WO 2008/1555 34 A2 number at the World Intellectual Property Organization). All the other authors declare that the research was conducted in the absence of any commercial or financial relationships that could be construed as a potential conflict of interest.

## Abbreviations

BDNF: brain-derived neurotrophic factor
BNN27: C17-spiroepoxy analog of DHEA
CHO: Chinese hamster ovary cell line
CYP17: cytochrome P450 17-hydroxylase/17,20-lyase
DHEA: dehydroepiandrosterone
ECD: extracellular domain
ERK: extracellular signal-regulated kinase
GAPDH: glyceraldehyde-3-phosphate dehydrogenase
HEK293: human embryonic kidney cell line 293
JNK: c-Jun N-terminal kinase
MEFs: mouse embryonic fibroblasts
NGF: nerve growth factor
NT-3: neurotrophin-3
p75KO: p75 receptor knockout
p75^NTR^: p75 neurotrophin receptor
PC12: pheochromacytoma cells
PEG-BNN27: polyethylene glycol amino resin-supported BNN27
RhoGDI: Rho GDP dissociation inhibitor
RIP2: receptor-interacting protein 2
STD NMR: saturation transfer difference nuclear magnetic resonance
TRAF6: TNF receptor-associated factor 6
Trk: tropomyosin related kinase
wt: wild type
