# Supplementary material for: BNN27, a 17-Spiroepoxy Steroid Derivative, Interacts With and Activates p75 Neurotrophin Receptor, Rescuing Cerebellar Granule Neurons from Apoptosis
Source: Front Pharmacol. 2016 Dec 26;7:512. doi: 10.3389/fphar.2016.00512 (PMC5183592; doi:10.3389/fphar.2016.00512)

Supplementary Figure Legends

Supplementary Figure 1. Predicted binding sites at the extracellular part of p75^{NTR} as defined by the generated site points

Application of SiteMap algorithm to the p75^{NTR} receptor identified five potential druggable binding sites. For visualization purposes the NGF homodimer is also depicted as obtained from the crystal complex (pdb:1SG1).

Site **1** located at the N-terminus of the protein coinciding with CRD1, site **2** is located at the CRD2 region at the opposite side of the NGF epitope interfacial region, site **3** coinciding with CRD3 region lays in the interfacial region between the two NGF binding epitopes (assigned as Site I and II in He and Garcia, 2004), and site **4** expanding at the lower NGF binding epitope (assigned as Site II in He and Garcia, 2004) and lying in the interfacial region and site **5** located at the C-terminal region, including amino acids of CRD4, facing at the opposite side of the interfacial region (depicted as surface in the Figure).

Supplementary Figure 2. MD simulations of BNN27 (30 and 50 ns) at the p75^{NTR} monomer

Snapshot from the MD simulations performed at the p75NTR with BNN27. Simulations were run with 5 BNN27 molecules for 50ns (A) and 10 BNN27 molecules for 30 ns (B). In both simulations the ligand stabilizes interactions although using one of its polar edges with amino acids of CRD4 at site **5** (i.e. hydrogen bonding with Thr140 and Gln148).

Supplementary Figure 3. *In silico* modeling of BNN27 binding at $p75^{NTR}$:2NGF complex

MD simulations (200 ns) of BNN27 at the $p75^{NTR}$:2NGF complex. (A) Initial state with BNN27 manually placed in the neighboring of the most potent predicted binding site of $p75^{NTR}$ located at the CRD4 region facing at the opposite side of the $p75^{NTR}$:2NGF interfacial region. (B) A representative 3D frame of the MD simulation at 154 ns (water-mediated interactions are omitted). During the simulation, the ligand migrates towards the $p75^{NTR}$:2NGF interfacial region and stabilized interactions with crucial contact residues of the $p75^{NTR}$:2NGF complex (NGF Arg^{114}A and $p75^{NTR}$ Cys^{136}). (C) 2D ligand interaction diagram of the simulation frame at 154 ns depicting the stabilized BNN27 interactions.

Supplementary Figure 4. *In silico* modeling of BNN27 binding at $p75^{NTR}$:proNGF modified complex

MD simulations (150 ns) of BNN27 at the $p75^{NTR}$:proNGF 2:2 complex with proNGF modified to model NGF homodimer. (**A**) A representative 3D frame of the MD simulation at 82 ns (water-mediated interactions are omitted). Already during the initial 40 ns, the ligand was spontaneously inserted at an interfacial region located at the $p75^{NTR}$ CRD1-CRD2 junction, "sandwiched" between the lipophilic residues NGF Phe49B and NGF Trp99B and contacts through H-bond with its 3-OH group $p75^{NTR}$ Asp41. The conserved salt bridge of $p75^{NTR}$ Asp41- NGF Lys88A is maintained. $p75^{NTR}$ receptor is colored in rainbow, chain A of NGF is colored in white and chain B in pink. (B) 2D simulation

interaction diagram depicting the BNN27 interactions stabilized for more than 15% of the simulation time.

Supplementary Figure 5. Sequence alignment between the crystallized human NGF (homodimer from pdb:1SG1) and the mature region of mouse proNGF (homodimer from pdb:3IJ2).

Application of Jalview s/w indicated 9 differentiation points between human-NGF and mouse-proNGF sequences.

Supplementary Figure 1

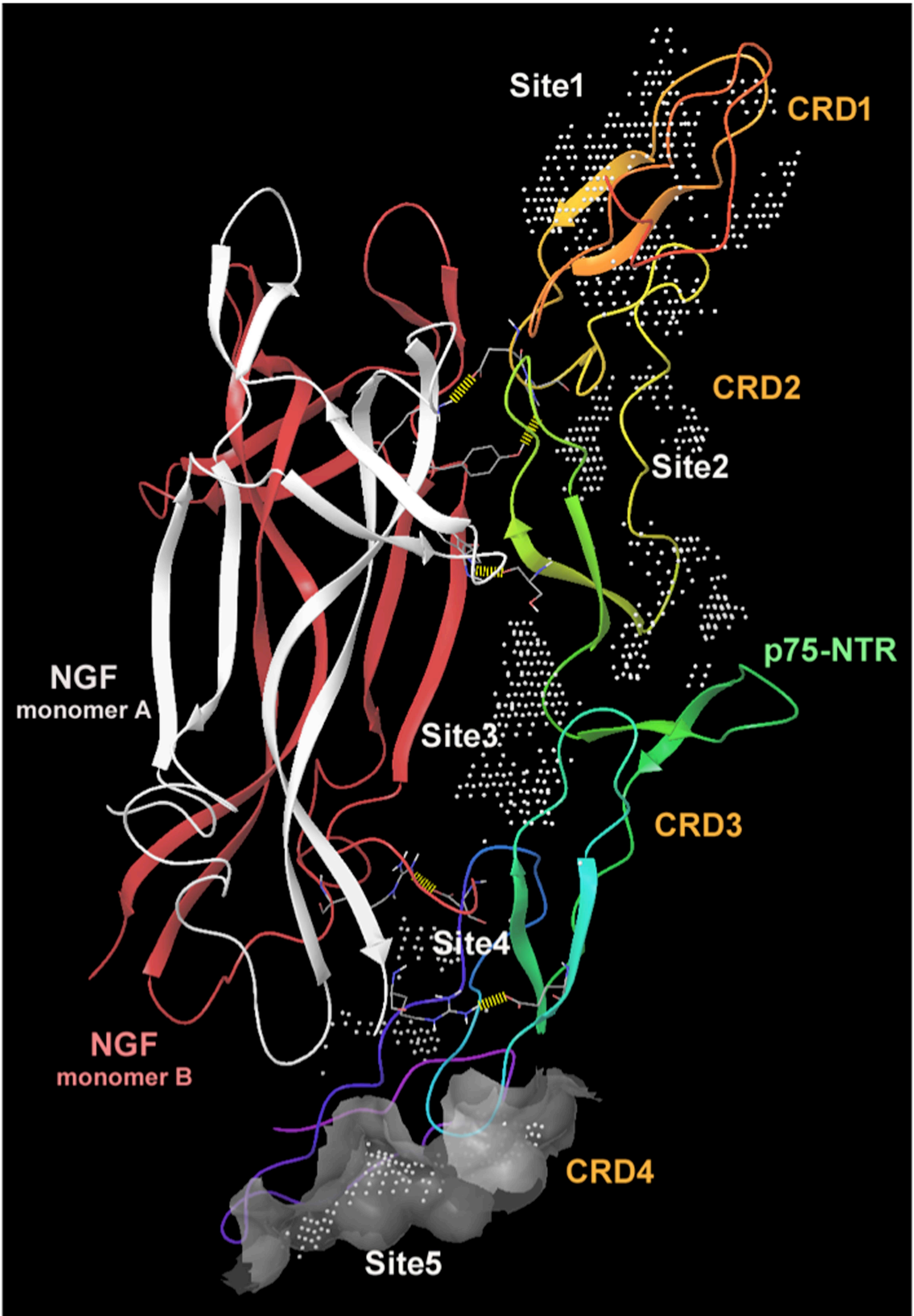

Supplementary Figure 2

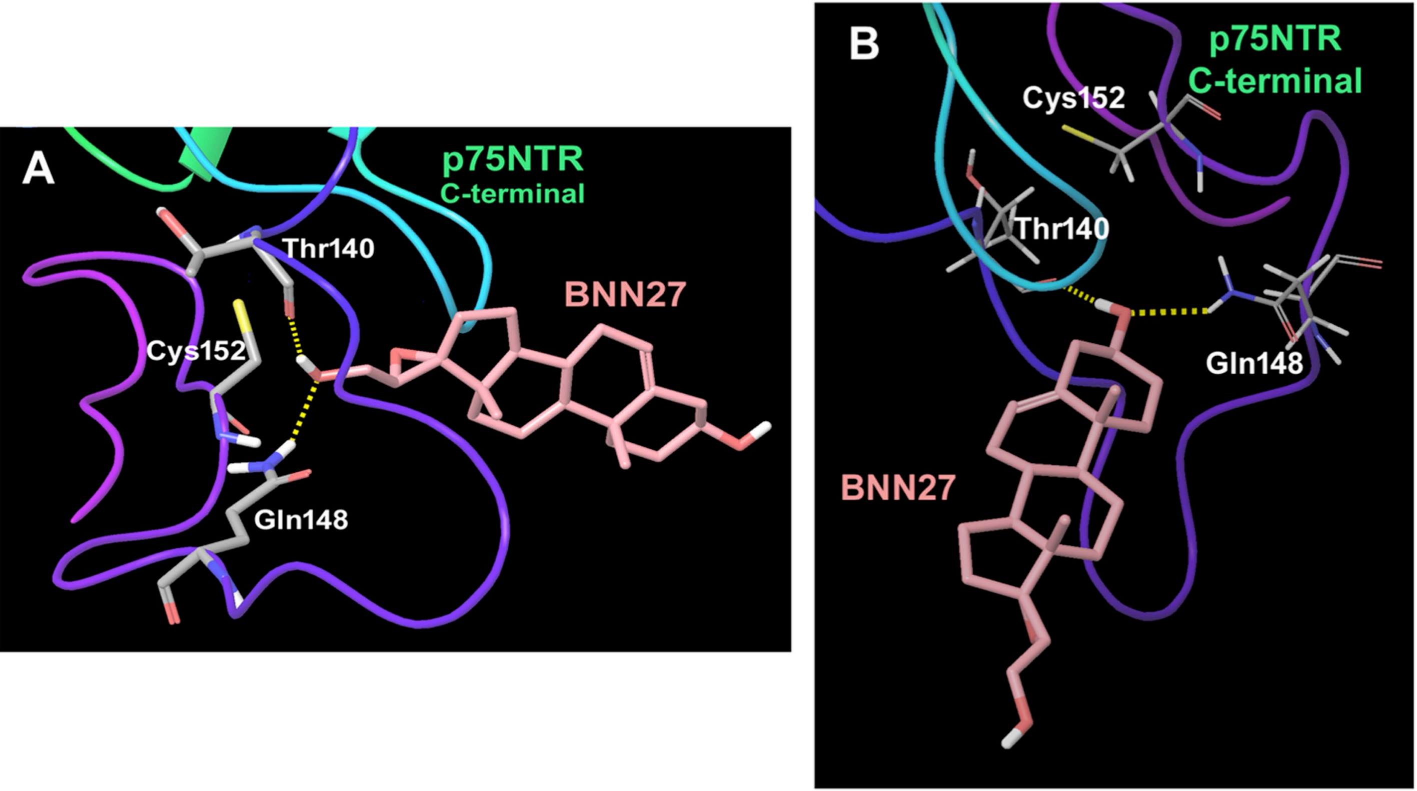

Supplementary Figure 3

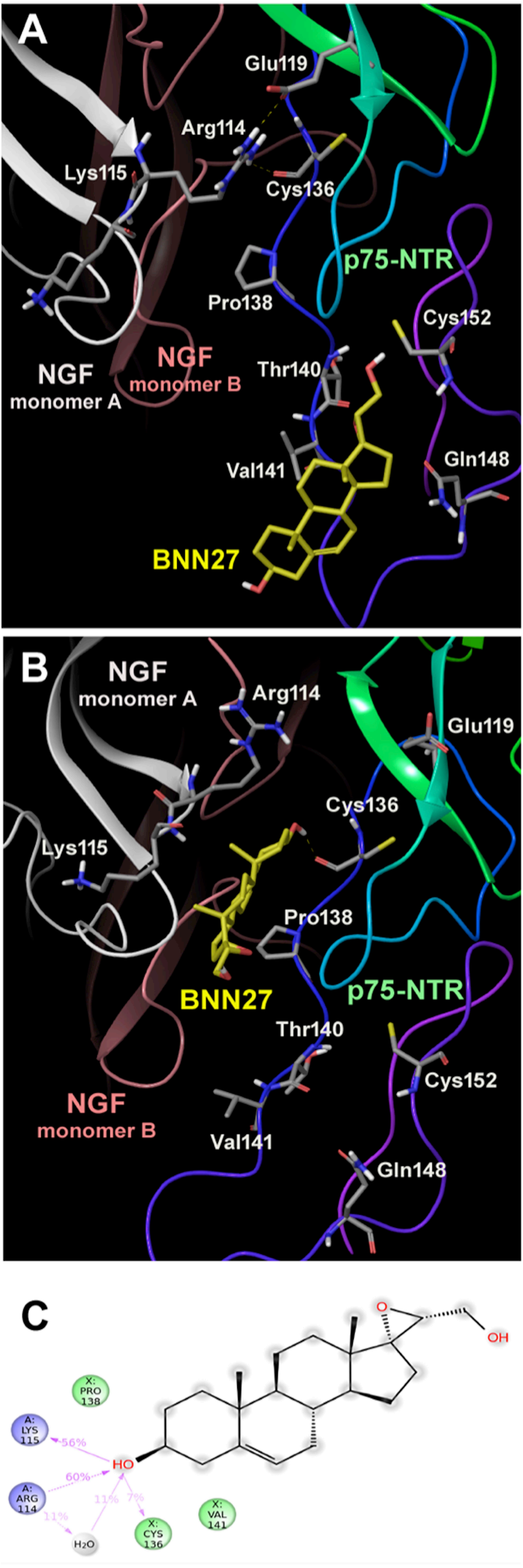

Supplementary Figure 4

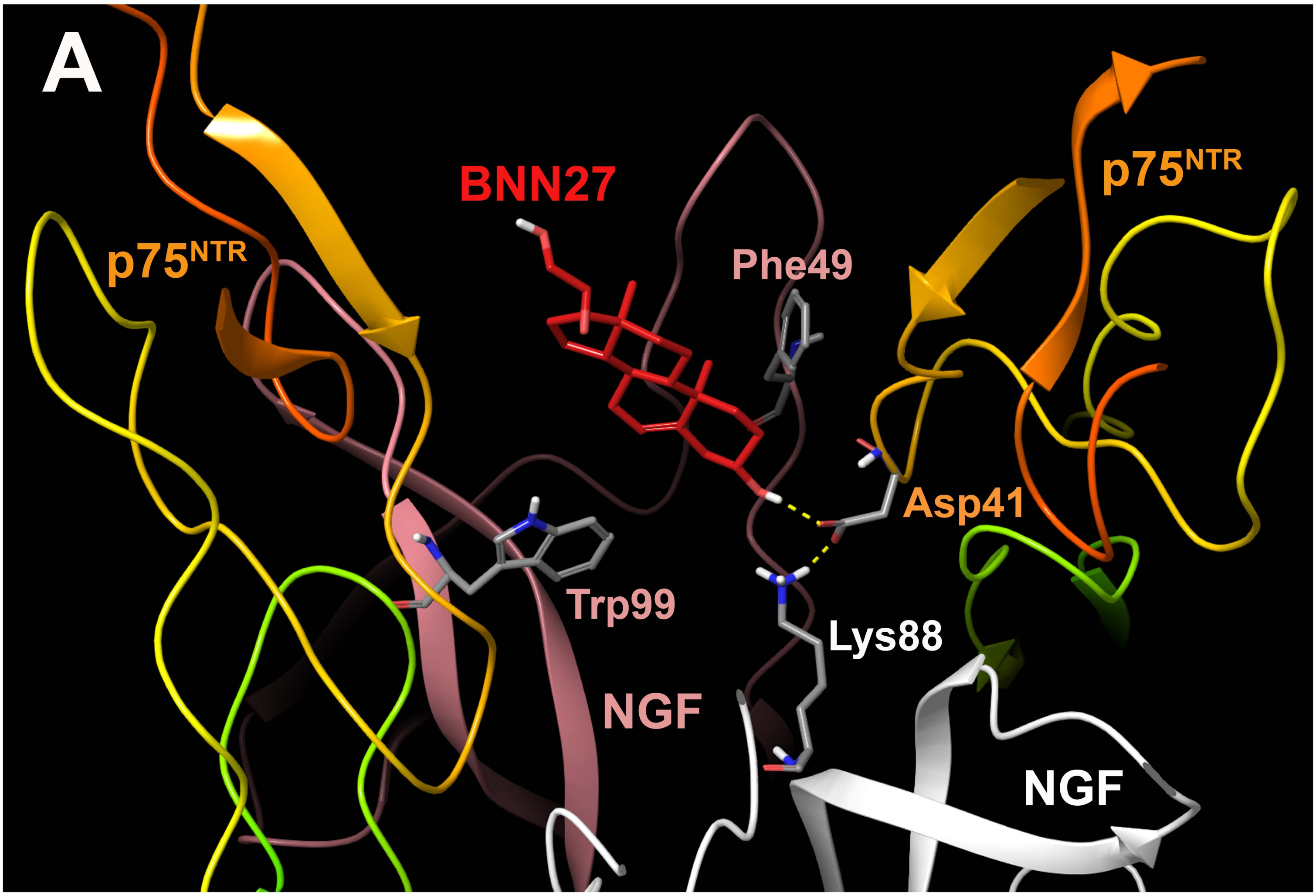

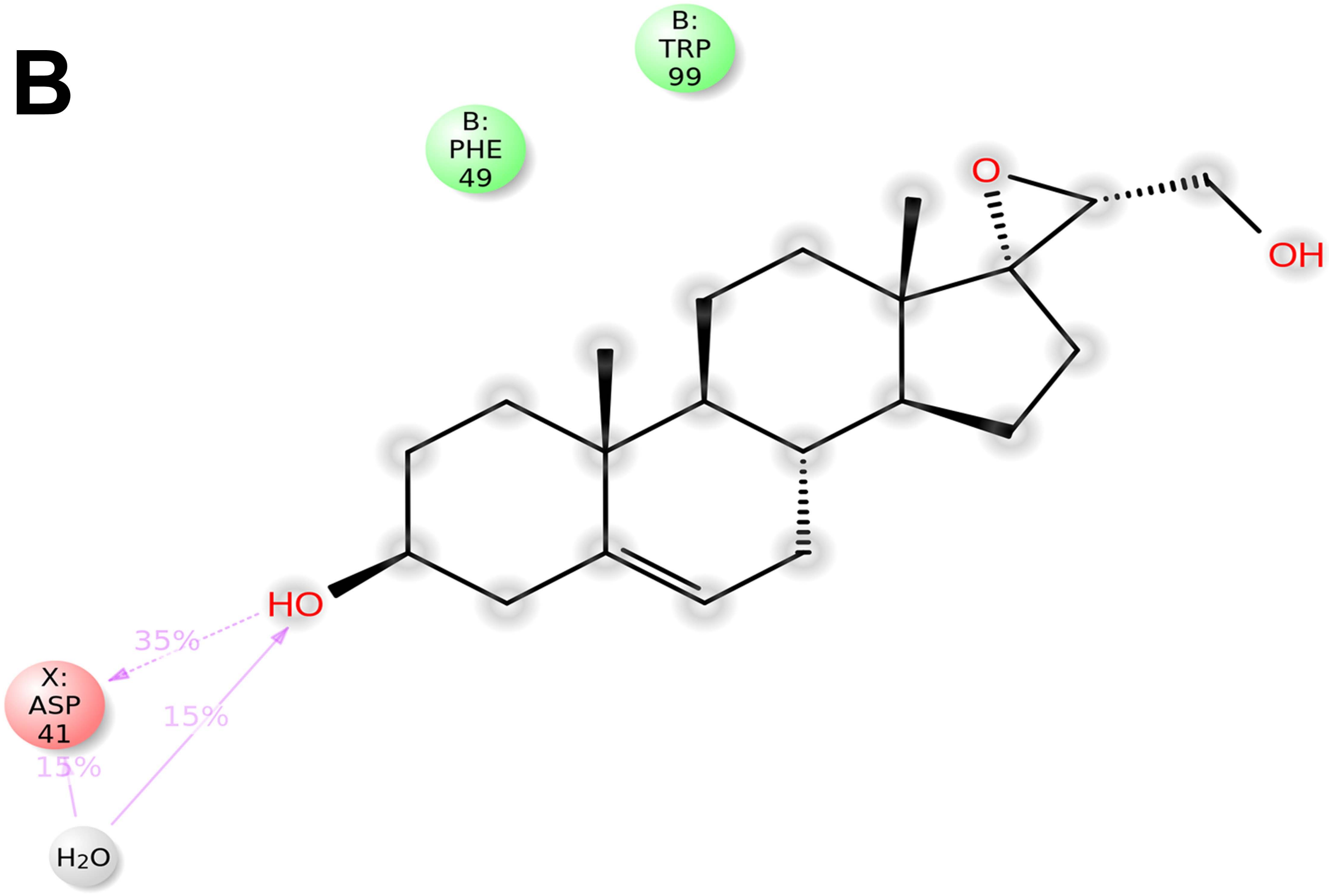

Supplementary Figure 5

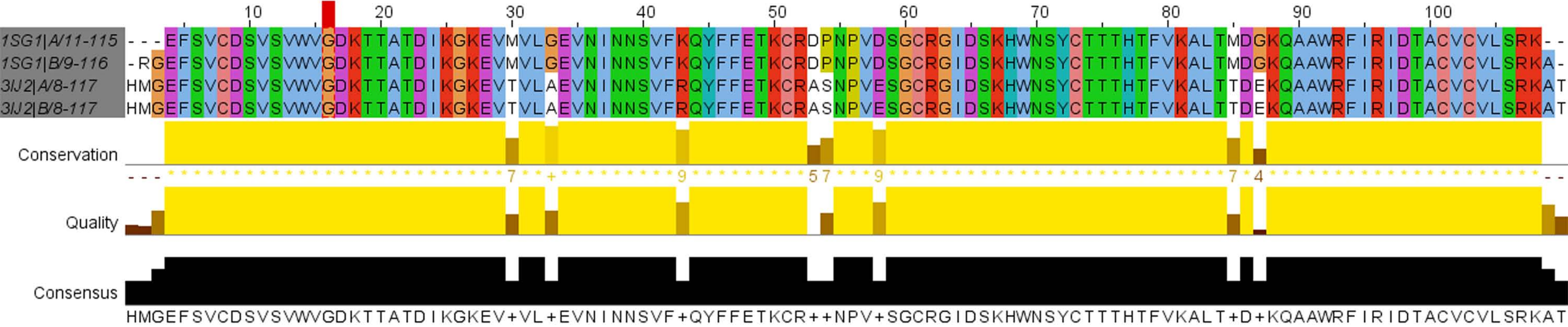

Supplement: Supplementary file 1 [file Data_Sheet_1.pdf]
